# Effect of the anti-parasitic compounds pyrvinium pamoate and artemisinin in enzymatic and culture assays: Data on the search for new anti-echinococcal drugs

**DOI:** 10.1016/j.dib.2020.106629

**Published:** 2020-12-09

**Authors:** Shigehiro Enkai, Hirokazu Kouguchi, Daniel Ken Inaoka, Takao Irie, Kinpei Yagi, Kiyoshi Kita

**Affiliations:** a School of Tropical Medicine and Global Health, Nagasaki University, 1-12-4 Sakamoto, Nagasaki 852-8523, Japan; b Department of Pediatrics, Teikyo University School of Medicine, 2-11-1 Kaga, Itabashi-ku, Tokyo 173-8605, Japan; c Department of Infectious Diseases, Hokkaido Institute of Public Health, N19 W12, Kita-Ku, Sapporo, Hokkaido 060-0819, Japan; d Department of Molecular Infection Dynamics, Shionogi Global Infectious Diseases Division, Institute of Tropical Medicine (NEKKEN), Nagasaki University, Nagasaki, 852-8523, Japan; e Laboratory of Veterinary Parasitic Diseases, Department of Veterinary Sciences, Faculty of Agriculture, University of Miyazaki, 1-1 Gakuen-Kibanadai Nishi, Miyazaki 889-2192, Japan; f Department of Host-Defense Biochemistry, Institute of Tropical Medicine (NEKKEN), Nagasaki University, 1-12-4 Sakamoto, Nagasaki 852-8523, Japan

**Keywords:** *Echinococcus multilocularis*, Echinococcosis, Atovaquone, Pyrvinium pamoate, Artemisinin, Drug target, Mitochondria

## Abstract

The dataset presented herein is related to a previous research article titled “Mitochondrial Complex III in Larval Stage of *Echinococcus multilocularis* as a Potential Chemotherapeutic Target and *in vivo* Efficacy of Atovaquone Against Primary Hydatid Cysts” [1]. In this report, data were collected by screening drugs for echinococcosis. We investigated the inhibitory activities of artemisinin and pyrvinium pamoate against the mitochondrial respiratory enzymes in *E. multilocularis* protoscoleces. Artemisinin did not inhibit mitochondrial complexes I, II, and III. However, pyrvinium pamoate inhibited complex I at 11 μM, although complexes II and III were not inhibited. In the culture assay, *E. multilocularis* protoscoleces were treated with atovaquone (ATV), rotenone, praziquantel, artemisinin, and pyrvinium pamoate at a final concentration of 50 µM in different culture media. The viability of protoscoleces was compared under aerobic and anaerobic conditions via culture experiments. The survival days of *E. multilocularis* protoscoleces were evaluated in the drug-treated group compared with those in the non-treated group. The results of these culture assays revealed that praziquantel and artemisinin did not eliminate the protoscoleces under both aerobic and anaerobic conditions. However, a stronger elimination ability was observed with the co-administration of praziquantel or artemisinin with ATV than with ATV alone under aerobic conditions. Pyrvinium pamoate completely killed protoscoleces at 5 and 7 days under aerobic and anaerobic conditions, respectively. Pyrvinium pamoate behaved identically to rotenone, the complex I inhibitor, in the culture treatment assay. The data serve as a reference for the development of novel anti-echinococcal drugs.

## Specifications Table

**Table:** 

Subject	Drug Discovery
Specific subject area	Mitochondrial respiratory system
Type of data	Figure and table
How data were acquired	IC_50_ of artemisinin and pyrvinium pamoate against the mitochondrial respiratory enzymes, and culture treatment assay using *E. multilocularis* protoscoleces
Data format	Raw Analyzed
Parameters for data collection	IC_50_ of artemisinin and pyrvinium pamoate against the mitochondrial respiratory enzymes in *E. multilocularis* protoscoleces was measured. The viability of protoscoleces was compared under aerobic and anaerobic conditions in culture experiments. Survival rate and days of *E. multilocularis* protoscoleces were evaluated in the drug-treated and non-treated groups.
Description of data collection	Inhibitory activities of artemisinin and pyrvinium pamoate against the mitochondrial respiratory enzymes were determined using a spectrophotometer (SHIMADZU UV-3000, Kyoto, Japan). In the culture, *E. multilocularis* protoscoleces were treated with atovaquone, rotenone, praziquantel, artemisinin, and pyrvinium pamoate at a final concentration of 50 µM in each culture medium. The viability of protoscoleces was determined by microscopic analysis of more than 170 protoscoleces per well for the ability to exclude trypan blue.
Data source location	Institution: Hokkaido Institute of Public Health City: Sapporo Country: Japan Latitude and longitude (and GPS coordinates) for collected samples/data: 43 °04′58.804′'N; 141 ° 19′59.769′'E.
Data accessibility	With the article
Related research article	S. Enkai, D.K. Inaoka, H. Kouguchi, T. Irie, K. Yagi, K. Kita, Mitochondrial complex III in larval stage of *Echinococcus multilocularis* as a potential chemotherapeutic target and *in vivo* efficacy of atovaquone against primary hydatid cysts, Parasitol. Int. 75 (2019) 102004. https://doi.org/10.1016/j.parint.2019.102004

## Value of the Data

• The data provide the inhibitory activity of artemisinin and pyrvinium pamoate against the mitochondrial respiratory chain of *E. multilocularis*. Artemisinin did not inhibit mitochondrial complexes I, II, and III in *E. multilocularis* protoscoleces. Pyrvinium pamoate inhibited the mitochondrial complex I activity of *E. multilocularis* protoscoleces at IC_50_ 11.3 μM.
• The data describe the efficacy of the combination of atovaquone with praziquantel or artemisinin against *E. multilocularis* protoscoleces in culture assays. The combined administration shortened the duration of parasite elimination compared with atovaquone alone under the aerobic conditions, but had no effect under anaerobic conditions.
• In culture experiments, pyrvinium pamoate, which inhibited mitochondrial complex I, completely eliminated *E. multilocularis* protoscoleces at 6 and 7 days under aerobic and anaerobic condition, respectively.

## 1 Data Description

**Fig. 1:**
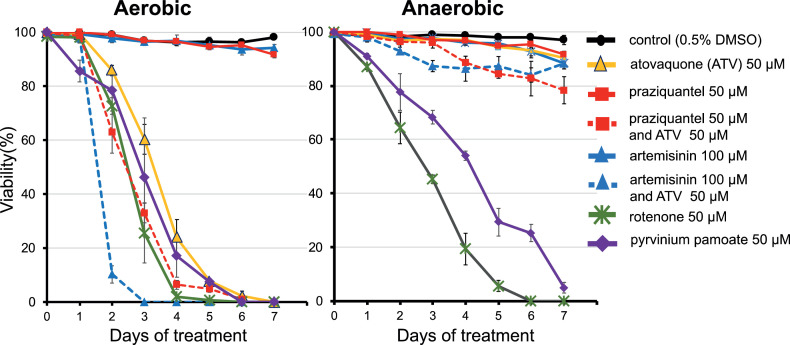
Culture under aerobic and anaerobic conditions (O2 < 0.3%); E. multilocularis protoscoleces were treated with atovaquone, rotenone, praziquantel, artemisinin, and pyrvinium pamoate at a final concentration of 50 µM in each culture medium. The viability of protoscoleces was evaluated as their ability to exclude trypan blue. The data are presented as mean and standard deviation of triplicate samples. Rotenone was added as a positive control for drug treatment as previously reported [1], [2]. The raw data tables are available as supplementary material.

The data in Table 1 show that pyrvinium pamoate inhibited mitochondrial complex I at IC_50_ 11.3 μM but did not inhibit complexes II and III (IC_50_ > 30 μM). Artemisinin did not block mitochondrial complexes I, II, and III (IC_50_ > 40 μM). Fig. 1 shows the anti-parasitic effect of 50 μM atovaquone (ATV), rotenone, praziquantel, artemisinin, pyrvinium pamoate, and combination chemotherapy of ATV and other drugs in a culture experiment using *E. multilocularis* protoscoleces. Praziquantel and artemisinin did not eliminate *E. multilocularis* protoscoleces under both aerobic and anaerobic conditions. Interestingly, under aerobic conditions, a stronger elimination ability was observed with the co-administration of praziquantel or artemisinin with ATV than with ATV alone. Pyrvinium pamoate killed all protoscoleces at 6 days under aerobic conditions and 95 % at 7 days under anaerobic conditions.

**Table 1:** The inhibitory effect of pyrvinium pamoate and artemisinin for the mitochondrial respiratory chain of E.multilocularis protoscoleces.

IC_50_ (μM)			
	NADH-ubiquinone reductase	Succinate-quinone reductase: SQR	Succinate-cytochrome *c* reductase
	(Complex I)	(Complex II)	(Complex II and III)
Pyrvinium pamoate	11 ± 0.3	> 30	> 30
Artemisinin	> 40	> 40	> 40

## 2 Experimental Design, Materials and Methods

### 2.1 Preparation of the mitochondrial fraction from E. multilocularis protoscoleces

All experiments were performed as described previously [1]. The infected cotton rats (*Sigmodon hispidus)* were sacrificed by isoflurane overdose to obtain the cyst tissues containing *E. multilocularis* (Nemuro strain) protoscoleces. The tissues were passed through a metal mesh and shredded completely. Thereafter, the tissues were repeatedly suspended and washed in a tall beaker to isolate protoscoleces using the difference in buoyancy between the protoscoleces and other tissue [1], [2]. The parasite materials were homogenized with a motor-driven homogenizer to prepare the mitochondrial fraction of *E. multilocularis* protoscoleces, while icing them. The homogenate was diluted with the mitochondrial preparation buffer (210 mM mannitol, 10 mM sucrose, 1 mM disodium EDTA, and 50 mM Tris-HCl; pH 7.5) supplemented with 10 mM sodium malonate to five times the volume of the original protoscolex sediment, and then centrifuged at 800 × *g* for 10 min (4  °C) to remove cell debris and nuclei. The supernatant was then centrifuged at 8,000 × *g* for 10 min (4 °C) to obtain the mitochondrial pellet. The pellet was re-suspended in mitochondrial preparation buffer (without malonate) and centrifuged at 8,000 × *g* for 10 min (4 °C). The enriched mitochondrial fraction was suspended in mitochondrial preparation buffer without malonate.

### 2.2 Enzyme assays and 50% inhibitory concentration (IC50) determination

All enzyme assays using mitochondrial fractions were performed in 0.5 or 1 mL reaction mixtures at 25 °C using an absorption spectrophotometer. Before the assay, the mitochondrial suspension was thawed at room temperature and refrozen in a deep freezer. The refrozen sample was thawed again after 1 h. This freezing and thawing process was performed before the assay to ensure that the mitochondrial membrane was permeable to the solutes. Without this process, NADH cannot pass through the mitochondrial membrane as a substrate. The reagents used in each assay were mixed with the reaction buffer (30 mM potassium phosphate and 1 mM MgCl_2_, pH 7.5). The final mitochondrial protein concentration was 50 μg/mL. NADH-decyl rhodoquinone reductase activity (complex I), succinate quinone reductase activity (complex II), and succinate-cytochrome *c* reductase activity (complexes II and III) were measured using a SHIMADZU spectrophotometer UV-3000 (Kyoto, Japan) as described previously [1], [2]. The IC_50_ values of pyrvinium pamoate and artemisinin were determined by calculating approximation lines from three or more points of anteroposterior concentration to the IC_50_ values.

### 2.3 Culture treatment of E. multilocularis protoscoleces

The metacestodes from one cotton rat at 4 months after infection with 50 eggs were full of mature protoscoleces and were enough to perform the culture experiment. Praziquantel alone cannot inhibit the mitochondrial respiratory system [3]. However, praziquantel can exert its effects in synergy with albendazole, although synergy with ATV has not been demonstrated [4]. The complementary culture experiment based on our previous report was performed to determine the efficacy of the combination therapy of ATV with praziquantel or artemisinin. Additionally, the efficacy of pyrvinium pamoate, which inhibited complex I of *E. multilocularis*, was ascertained in a culture assay under aerobic and anaerobic conditions. In this experiment, rotenone was used as the control to compare with previous report findings [1], [2].

The obtained protoscoleces were cultured in Connaught Medical Research Laboratories 1066 medium (Gibco, Grand Island, NY, USA) containing 23 mM 4-(2-hydroxyethyl)-1-piperazineethanesulfonic acid, 0.5% (w/v) D (+)-glucose, 0.4 mM sodium taurocholate (Wako Pure Chemical Industries), 0.5% (w/v) yeast extract (Difco Laboratories, Detroit, MI, USA), 57 mM sodium hydrogen carbonate, 2 mM L-glutamine (Gibco), 100 U/mL penicillin, and 100 μg/mL streptomycin (Gibco). Half of the medium was replaced on day 3. For anaerobic cultures, six-well plates were sealed in plastic containers with oxygen-detecting agents and oxygen scavengers (Aneromeito®, Nissui Pharmaceutical, Tokyo, Japan) to maintain the oxygen concentration under 0.3% at 37 °C. To assess the effect of chemical compounds against *E. multilocularis* protoscoleces, the parasites were treated with rotenone, praziquantel, artemisinin, pyrvinium pamoate, and ATV at a final concentration of 50 µM in the culture medium. The control group was supplemented with 0.5% (v/v) dimethyl sulfoxide (DMSO), and all conditions were assayed in triplicate. The viability of protoscoleces was determined by microscopic observation of more than 170 protoscoleces per well using the trypan blue exclusion test. The protoscoleces were observed daily for 7 consequent days, and the efficacy of the treatment was evaluated by the duration of parasite elimination based on previous reports [1], [2], [5], [6].

## Ethics Statement

The procedures in this manuscript are in strict accordance with the National Institutes of Health guide for the Care and Use of Laboratory animals. Furthermore, the protocol for animal experiments was approved by the ethics committee of the Hokkaido Institute of Public Health (permit numbers: K25-2 and K29-4).

## Declaration of Competing Interest

The authors declare that they have no known competing financial interests or personal relationships that have, or could be perceived to have, influenced the work reported in this article.

## Appendix Supplementary materials

Image, application 1
